# The Immunity Protection of Central Nervous System Induced by Pseudorabies Virus DelgI/gE/TK in Mice

**DOI:** 10.3389/fmicb.2022.862907

**Published:** 2022-03-25

**Authors:** Lei Xu, Jian-feng Wei, Jun Zhao, Si-yao Xu, Feng-qin Lee, Min-cai Nie, Zhi-wen Xu, Yuan-cheng Zhou, Ling Zhu

**Affiliations:** ^1^Key Laboratory of Animal Diseases and Human Health of Sichuan Province, College of Veterinary Medicine, Sichuan Agricultural University, Chengdu, China; ^2^Livestock and Poultry Biological Products Key Laboratory of Sichuan Province, Sichuan Animal Science Academy, Chengdu, China; ^3^Animal Breeding and Genetics Key Laboratory of Sichuan Province, Sichuan Animal Science Academy, Chengdu, China

**Keywords:** pseudorabies virus, neurological damage, intestinal immunity, blood–brain barrier, inflammation

## Abstract

Based on a variant strain, we constructed a gE/gI/TK-deleted pseudorabies virus (PRV). A total of 18 female mice were randomized to a vaccination group to receive PRV XJ delgE/gI/TK, a vehicle group to receive Dulbecco’s modified Eagle’s medium, and a mock group to confirm the protection of PRV delgE/gI/TK on the central nervous system in mice. Subsequently, the vaccination and vehicle groups were infected with PRV XJ. The mice in the vehicle group showed more severe neurological symptoms and higher viral loads than those in the vaccination group. The exudation of Evans blue and the expression of tight junction protein showed no difference in all groups. HE staining showed vacuolar neuronal degeneration in the vehicle group brain, but no tissue lesions were observed in the vaccination group. TNF-α, IL-6, and synuclein were upregulated in the brain of mice in the vehicle group, while those were inhibited among mice in the vaccination group. IFN-β, IFN-γ, ISG15, Mx1, and OAS1 showed no difference in the brain between the vaccination and vehicle groups. In addition, TNF-α and IL-6 were inhibited, and antiviral factors were increased in the intestine of the mice in the vaccination group compared to those in the vehicle group. Our study showed that PRV XJ delgE/gI/TK inhibited neurological damage and the inflammation of the intestine and brain induced by PRV and activated the innate immunity of the intestine.

## Introduction

Pseudorabies virus (PRV), also known as Aujeszky’s disease virus, is a member of *Herpesviridae*, subfamily *Alphaherpesvirinae*, and genus *Varicellovirus* (Pomeranz et al., 2005). This disease was controlled and eradicated from the swine population in most parts of China before 2011 using Bartha-K61 strain inoculation of PRV-infected swine herds. Nevertheless, since October 2011, pseudorabies outbreaks among the Bartha-K61-immunized swine population have spread quickly in China, which caused significant economic losses to the swine industry. The virus genome analysis showed that the re-emerging PRV belongs to a variant strain of genotype-2, and the Bartha-K61 vaccine cannot provide complete protection against the challenge with the emerging PRV variants. Compared with the PRV classical strain, the PRV variants showed stronger virulence, infectious ability, faster transmission speed, more serious clinical symptoms, and higher mortality rates. In a study by Yang et al. (2016) porcine infected with the variant PRV exhibited more serious respiratory symptoms and neurological signs, more severe damage to organs, and more extensive virus distribution and viral loads in different organs compared to classical PRV. Various animals are susceptible to PRV, including cats, rabbits, dogs, cattle, sheep, and goats, but only porcine are the natural host and reservoir (Zhang et al., 2015; He et al., 2019; Laval and Enquist, 2020). PRV infection causes high mortality in young piglets, growth retardation in growing pigs, and reproductive failure in sow. The major clinical symptoms include fever, itchiness, respiratory symptoms, ataxia, and tetany (An et al., 2013; Wu et al., 2013; Luo et al., 2014). PRV has been thought to cause diseases only in animals. However, recent evidence has shown that PRV might cause encephalitis in humans, which has severe clinical manifestations, including fever, sweating, weakness, status epilepticus, and even death, and the prognosis of patients is extremely poor (Wang et al., 2019a; Yang et al., 2019; Ou et al., 2020), which poses a serious threat to public health and safety. In late 2015, a PRV strain which caused a large number of deaths of piglets was isolated and named PRV XJ. Our study constructed a gE/gI/TK-deleted PRV based on the PRV XJ strain and evaluated the central nervous system protection of PRV XJ delgE/gI/TK and the intestinal immunity induced by PRV XJ delgE/gI/TK.

## Materials and Methods

### Virus and Cells

The PRV XJ strain (GenBank accession no. MW893682), a variant strain, was isolated and identified by our laboratory. PRV XJ del gI/gE, in which the gI and gE genes are deleted, was constructed by our laboratory. All the viruses were propagated in BHK-21 cells cultured in Dulbecco’s modified Eagle’s medium (DMEM) supplemented with 10% (v/v) fetal bovine serum.

### Generation of PRV XJ DelgE/gI/TK

Briefly, single guide RNAs (sgRNA) targeting the TK gene were designed with the CRISPR design tool^1^ (Table 1) and cloned into the lenticrispr-V2 vector, which was designated TK-sgRNA. The TK-sgRNA was verified by sequencing. BHK-21 cells were co-transfected with 1.5 μg of PUC57-TK-RED and 1.5 μg of CRISPR/Cas9 plasmid lentivrispr-V2 carrying the two sgRNAs targeting the TK gene. The BHK-21 cells were inoculated with PRV XJ del gI/gE when the red fluorescence proteins were expressed. When a distinct cytopathic effect was observed, the cultures were collected and subjected to six rounds of plaque purification. The gE/gI/TK-deleted virus was verified by PCR using gB, gE, and TK primers, respectively (Table 1). Finally, the purified gE/gI/TK-deleted virus was designated XJ del gE/gI/TK.

**TABLE 1:** Primers for recombinant virus construction and detection.

Primer name	Sequence	Size/position
gE-F	ATCTGGACGTTCCTGCCC	534 bp
gE-R	GTAGATGCAGGGCTCGTACA	
gB-F	CGGCAAGTGCGTCTCCAAG	255 bp
gB-R	AGGGCGAAGGAGTCGTAGGG	
TK-F	CATCCTCCGGATCTACCTCGACGGC	742 bp
TK-R	CACACCCCCATCTCCGACGTGAAGG	
sgRNA1	CTCGACGGCGCCTACGGCAC	22–41
	GTGCCGTAGGCGCCGTCGAG	
sgRNA1	GCCGCGTACGGCGACCACATC	902–921
	GATGTGGTCGCCGTACGCGG	
gE-sF	CTTCCACTCGCAGCTCTTCT	165 bp
gE-sR	TAGATGCAGGGCTCGTACAC	

### One-Step Growth Kinetics

One-step growth kinetics was conducted to compare the growth kinetics of PRV XJ delgE/gI/TK with the parental virus PRV XJ. The BHK-21 cells were infected with the virus at a multiplicity of infection of 1. The cells were harvested at successive intervals after infection and stored at –80°C. A virus one-step growth curve was drawn based on 50% tissue culture infectious dose (TCID_50_).

### Animals and Experiment Design

Eighteen female Kunming mice were divided randomly into three groups (vaccination group, vehicle group, and mock group) with six mice in each group. All mice had free access to food and water and were kept at room temperature (23 ± 1.5°C). The mice in the vaccination group were intramuscularly injected with 10^6^ TCID_50_ PRV XJ delgI/gE/TK. Furthermore, booster vaccinations were performed at week 2. The mice in the vehicle and mock groups were intramuscularly injected with DMEM. Moreover, the mice in the vaccination and vehicle groups were challenged with 10^4^ TCID_50_ PRV XJ strain *via* intramuscular injection at week 2 after the booster vaccinations. Furthermore, the mice in the mock group were injected with DMEM. All mice were weighed and recorded after the challenge. Determination of the mean time to death and clinical evaluation were performed as described earlier (Sehl et al., 2020). All animal experiments were conducted in accordance with the guidelines of the local animal welfare bodies and the Sichuan Agricultural University Ethics Committee (SYXK2019-187).

### Evans Blue Extravasation

The permeability of the blood–brain barrier was determined using Evans blue dye; 2% Evans blue (2 ml/kg) was injected into the tail vein of the mice before they were sacrificed. The brain was dissected, weighed, homogenized with 50% trichloroacetic acid, and centrifuged (12,000 *g*, 15 min). The Evans blue contents of the supernatant were measured, based on absorbance at 620 nm, with a spectrophotometer and were calculated according to a standard curve.

### Quantitative Real-Time PCR Assay

The fresh intestine and brain of mice were dissected to analyze the expression of inflammation factors and antiviral factors in the intestine and brain. According to the manufacturer’s protocols, total RNA was extracted from the mice’s brain and intestine using RNAiso Plus. RNA concentration and purity were measured by ScanDrop using the A260 value and the ratio of A260/280, respectively. Reverse transcription reactions were performed using PrimeScript RT Kit. Quantitative RT-PCR was carried out on Roche Lightcycler96 instrument using TB Green Premix Ex Taq according to the manufacturer’s instructions. The forward and reverse primer sequences for each gene are provided in Table 2. Gene expression was quantified using the 2^–ΔΔCT^ method.

**TABLE 2:** Primers for qRT-PCR.

Primer	Sequence
gE-F	CTTCCACTCGCAGCTCTTCT
gE-R	TAGATGCAGGGCTCGTACAC
β-actin-F	CATCCGTAAAGACCTCTATGCCAAC
β-actin-R	ATGGAGCCACCGATCCACA
Occludin-F	CCTCCAATGGCAAAGTGAAT
Occludin-R	CTCCCCACCTGTCGTGTAGT
TNF-α-F	ATCCGCGACGTGGAACTG
TNF-α-R	ACCGCCTGGAGTTCTGGAA
IFN-γ-F	TCAAGTGGCATAGATGTGGAAGAA
IFN-γ-R	TGGCTCTGCAGGATTTTCATG
IL-6-F	TTGCCTTCTTGGGACTGATG
IL-6-R	ATTGCCATTGCACAACTCTT
IFN-β-F	GACGTGGGAGATGTCCTCAAC
IFN-β-R	GGTACCTTTGCACCCTCCAGTA
ISG15-F	CTCCTTAATTCCAGGGGACCT
ISG15-R	CGTCATGGAGTTAGTCACGG
Mx1-F	ACCAGGGTGGCTGTAGG
Mx1-R	CAGGTTGGGCATCACAT
OAS1-F	CATCCAGGAAATTCGGAGACAG
OAS1-R	GGCAGGACATCAAACTCCACCTC

Total DNA was extracted from different tissues of the mice using a universal genomic DNA kit. The viral loads in tissue samples from the challenged animals were determined with the qRT-PCR assay for the PRV gE gene using gE-specific primer (Table 1). The gene copy number for each sample was expressed as log10 copies per gram of tissue sample.

### Histopathology and Immunohistochemistry

The tissues were dissected, collected, and fixed in 4% paraformaldehyde for at least 72 h. The fixed tissues were embedded in paraffin wax and cut into 4-μm sections. The tissue sections were subjected to histopathological analysis by staining with hematoxylin and eosin.

Immunostaining was performed according to the streptavidin–biotin–peroxidase complex (SABC) immunoprecipitation kit. In brief, the sections were deparaffinized and rehydrated using xylene and graded concentrations of alcohol. Endogenous peroxidase activity was inhibited with 3% hydrogen peroxide for 15 min at room temperature. Then, the antigen epitope was subsequently unmasked using a citrate buffer *via* incubation with 100°C water bath for 15 min. Non-specific antigens were blocked with 5% bovine serum albumin (BSA). The sections were incubated with Syn1 (A17362, Abclonal, 1:200) overnight at 4°C in a moist chamber and rinsed three times with PBS. Then, the sections were incubated with a biotinylated secondary antibody for 30 min, followed by incubation with the SABC for 30 min. The sections were visualized using 3,3-diaminobenzidine and subsequently counterstained with hematoxylin for better visualization. The tissue sections were viewed with an Eclipse 50i microscope equipped with a camera. Images were captured using NIS-Elements 2.30 software.

### Western Blotting

Brain proteins were harvested and homogenized in lysis buffer containing protease inhibitor phenylmethanesulfony fluoride. The protein concentration was determined with the BCA Protein Assay Kit. Briefly, equal amounts of total protein were resolved by sodium dodecyl sulfate–polyacrylamide gel electrophoresis and then transferred onto a polyvinylidene fluoride membrane. The membrane blots were saturated with 5% BSA in phosphate-buffered saline with Tween 20 (PBST) for 2 h at room temperature and then incubated overnight at 4°C with primary antibodies against Syn1 (A17362, Abclonal, 1:1,000), β-actin (AC026, Abclonal, 1:50,000), and occludin (A2601, Abclonal, 1:1,000). After incubation, the membrane was washed three times with PBST and incubated with HRP Goat Anti-Rabbit IgG (H + L) (AS014, Abclonal, 1:10,000). The signals were visualized with SuperSignal™ West Pico Plus Chemiluminescent Substrate. The gray intensity of proteins was measured using ImageJ software.

### Enzyme-Linked Immunosorbent Assay

Blood samples were collected at 0, 7, 14, 21, and 28 days post-vaccination (dpv). The serum was separated by centrifugation after coagulation, and PRV-specific gB antibodies in serum were detected using ELISA kits (IDEXX, Bern, Switzerland) according to the manufacturer’s directions. The optical density at 650 nm was measured by a microplate reader (Bio-Rad, Hercules, CA, United States).

### Statistical Analysis

Statistical analysis was undertaken by one-way analysis of variance with GraphPad 7.04 software. All results were expressed as mean ± standard deviation from at least three replicates and were representative of three independent experiments. The value of *P* < 0.05 was considered statistically significant.

## Results

### Construction and Characterization of PRV XJ Del gE/gI/TK

**FIGURE 1:**
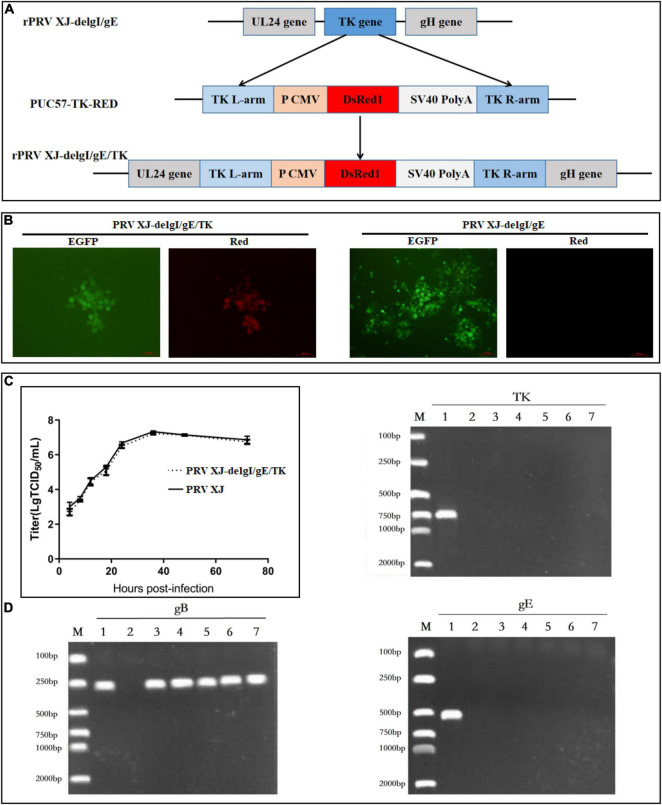
Construction and identification of PRV XJ delgE/gI/TK. **(A)** Schematic diagram of the construction for PRV XJ delgE/gI/TK. **(B)** Identification of rescued pseudorabies virus (PRV) from the distinct cytopathic effect cells with green and red fluorescence under the fluorescence microscope. **(C)** One-step growth curves. The BHK-21 cells were infected with PRV XJ or PRV XJ delgE/gI/TK at multiplicity of infection = 1. At designated time points, the cells were harvested, and the viral titers were measured. **(D)** gE/gI/TK deletion was verified in PRV XJ delgE/gI/TK genome by PCR. M, DL 2000 Marker; 1, PRV XJ; 2, H_2_O; 3–7, F6–F10.

The gE/gI/TK-deleted candidate strain was constructed with homologous recombination and CRISPR/Cas9 system, as shown in Figure 1A. Following the co-transfection of PUC57-TK-RED and the CRISPR/Cas9-TK-sgRNA plasmid, the BHK-21 cells were inoculated with PRV XJ del gE/gI. Then, the gE/gI/TK-deleted PRV plaques were isolated by plaque purification (Figure 1B). The gE-, TK-, and gB-specific primers were used to identify the gene deletion strain. The specific PCR products of gE and TK were not observed in PRV XJ delgE/gI/TK, whereas the gB gene could be detected (Figure 1C), which indicated that the PRV XJ delgE/gI/TK virus was constructed successfully. The reconstituted PRV XJ delgE/gI/TK growth features were analogous to that of the parental PRV XJ in BHK-21 cells (Figure 1D).

### PRV XJ DelgE/gI/TK Protects Mice Against Variant PRV XJ Strain

**FIGURE 2:**
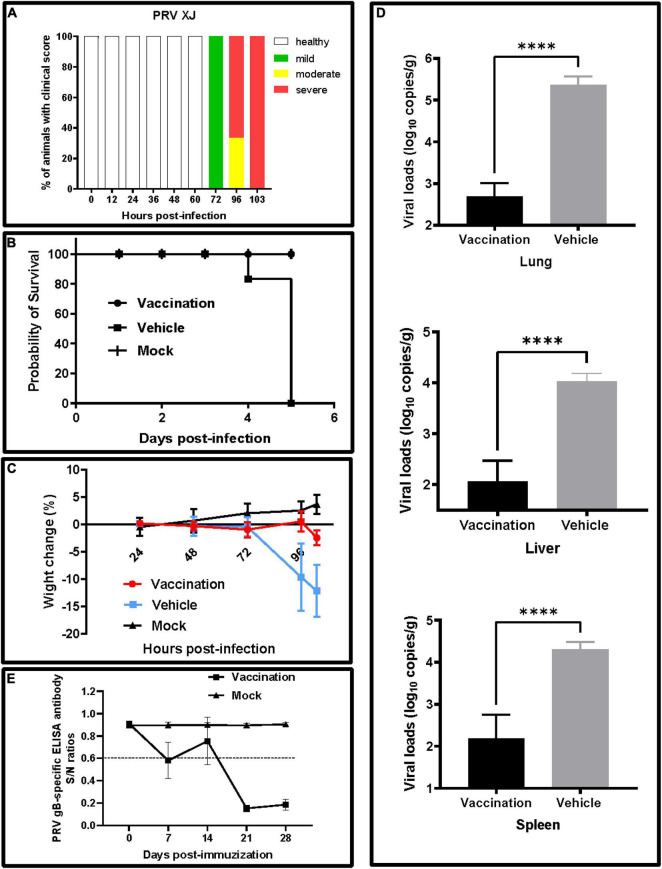
Clinical signs and viral loads of experimental mice. **(A)** Clinical score. The frequency of sick animals infected with pseudorabies virus (PRV) XJ. Based on a scoring system (Supplementary Table 1), the animals were categorized into either healthy (white) and mildly (green), moderately (yellow), or severely affected (red). **(B)** Survival curves for mice after a virulent challenge with PRV XJ. The survival percentages were presented as a Kaplan–Meier plot (*n* = 6 per group). **(C)** Weight change of mice in each group after immunization and challenge. **(D)** The viral DNA loads in the lung, liver, and spleen were determined by qRT-PCR. The viral DNA copy numbers were measured with specific primers for the gE gene. **(E)** Development of PRV gB-specific antibody. ****p < 0.0001.

At 3 days post-infection with the PRV XJ strain, all mice of the vehicle group showed clinical symptoms, including curved back, ruffled fur, pruritus, auto-mutilation, and dyspnea, and died at 5 days post-infection (Figures 2A,B). All mice in the vaccination and mock groups survived throughout the experiments, and no clinical signs were observed. Before the challenge, there were no significant differences in the weight gain of mice in each group. After the challenge, the mice in the vehicle group lost weight, but the mice in the vaccination group inoculated with PRV XJ delgE/gI/TK had weight changes similar to those in the mock group (Figure 2C). The viral loads were monitored in different tissues using qRT-PCR. The viral genome could be detected in the lung, spleen, and liver from the vaccination and vehicle groups. All tissue viral loads of the vaccination group were lower than those of the vehicle group (Figure 2D). The gB-specific antibodies of the vaccinated group were detected at 7 dpv. Then, the gB antibody levels decreased at 14 dpv. Finally, the gB antibodies levels increased again when booster vaccinations were performed and kept steadily increasing until 28 dpv. On the contrary, the gB-specific antibodies failed to be detected in the mock group (Figure 2E).

### PRV XJ Strain Infections Had No Effects on Blood–Brain Barriers in Mice

**FIGURE 3:**
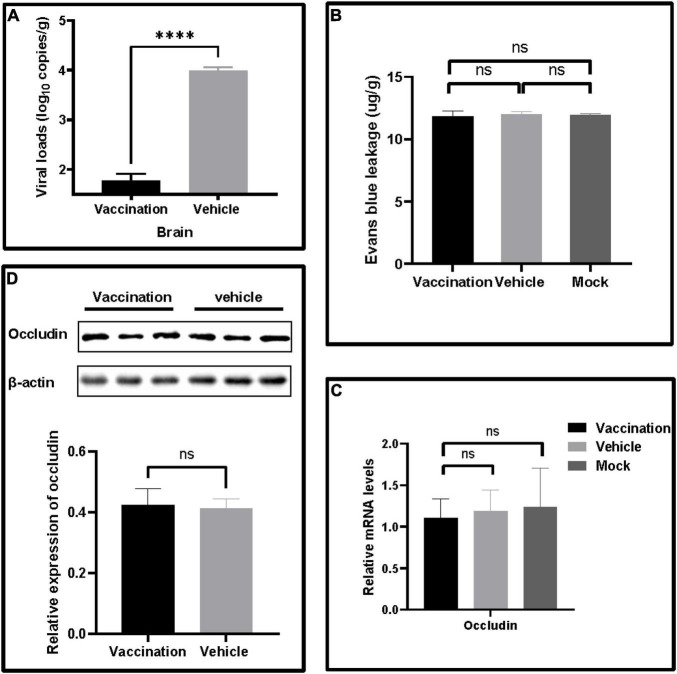
The effects on blood–brain barrier leakage after pseudorabies virus XJ challenge. **(A)** The viral DNA loads in the brain were determined by qRT-PCR. **(B)** Quantitative analysis of Evans blue dye extravasation. **(C)** The fold changes normalized with β-actin as an internal control of occludin mRNA levels in the brain after the challenge. **(D)** Western blot analysis and relative quantification of the band density of occludin. Data represent mean ± SD. ****p < 0.0001.

The viral loads of the brain were determined to confirm the protection of PRV XJ delgE/gI/TK on the central nervous system. The brain viral loads of the vaccination group were significantly lower than those of the vehicle group (Figure 3A). Furthermore, all mice were injected with Evans blue dye by tail vein injection to examine whether the PRV XJ delgE/gI/TK protected the blood–brain barrier permeability of mice. Evans blue was not detected in these mice (Figure 3B). Similarly, the tight junction protein occludin mRNA and the occludin protein levels were detected. The occludin mRNA levels of all mice brains showed no difference (Figure 3C). The occludin expression was no different in all groups (Figure 3D). These results altogether suggested that PRV XJ strain infections did not affect the mice’s blood–brain barrier permeability.

### PRV XJ DelgE/gI/TK Protects the Central Nervous System From Neurological Damage Induced by PRV XJ

**FIGURE 4:**
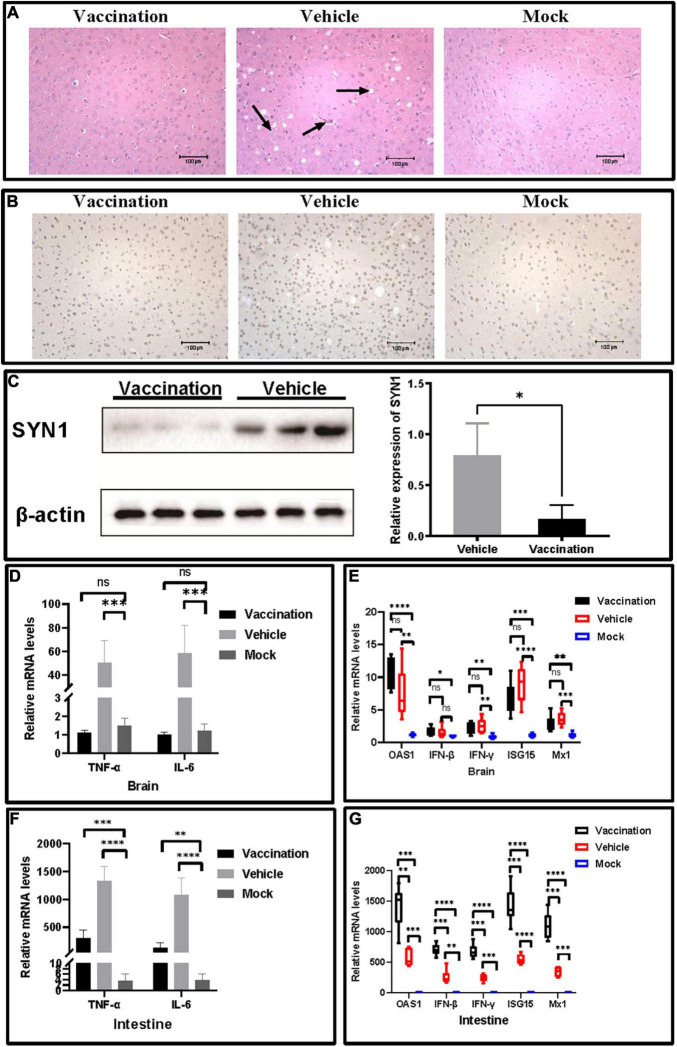
The protective effects of pseudorabies virus XJ delgE/gI/TK in mice brain. **(A)** Histopathological observations of mice brain injected with the indicated viral strain or Dulbecco’s modified Eagle’s medium. The arrows from the left to the right show nuclear cleavage, neuron phagocytosis, and vacuolar neuronal degeneration in the brain, respectively (hematoxylin and eosin staining, × 200 magnification). **(B)** Immunohistochemistry detection of SYN1 in mice. Comparison of the SYN1 expression in mice brain using immunohistochemistry. **(C)** Western blot analysis and relative quantification of the band density of SYN1. **(D)** The fold change of TNF-α and IL-6 in the brain was determined by qRT-PCR. **(E)** The fold change of TNF-α and IL-6 in the intestine was determined by qRT-PCR. **(F)** The fold change of IFN-β, IFN-γ, ISG15, Mx1, and OAS1 in the brain was determined by qRT-PCR. **(G)** The fold change of IFN-β, IFN-γ, ISG15, Mx1, and OAS1 in the intestine was determined by qRT-PCR. Data are presented as mean ± SD (*n* = 6). *P < 0.05, ^**^P < 0.01, ***P < 0.001, ****P < 0.0001.

To investigate the protection of PRV XJ delgE/gI/TK on the central nervous system in mice, we measured and compared the neurological damage in the brain of a different group. The histopathological examination showed vacuolar neuronal degeneration, neuron phagocytosis, and nuclear cleavage in the vehicle group, and no histopathological changes were observed in the vaccination group (Figure 4A). The expression of SYN1 protein in the brain was evaluated by immunohistochemistry. The brain of the vehicle group exhibited more SYN1 expression than the vaccination group (Figure 4B). Furthermore, Western blotting confirmed those results (Figure 4C). Those results suggested that PRV XJ delgE/gI/TK protected the central nervous system from neurological damage induced by PRV XJ. In addition, the pro-inflammatory and antiviral factor mRNA levels in the brain and intestine were determined. In the brain, the TNF-α and IL-6 mRNA levels of the vaccination group were significantly decreased compared with the vehicle group and showed no difference between the vaccination and mock groups, and the mRNA levels of IFN-β, IFN-γ, ISG15, Mx1, and OAS1 showed no difference between the vaccination group and the vehicle group but were significantly increased compared with those in the brain of mice in the mock group (Figures 4D,F). In the intestine, TNF-α and IL-6 were inhibited in the vaccination group compared with the vehicle group, and TNF-α and IL-6 were increased in the vaccination and vehicle groups compared with the mock group (Figure 4E). The antiviral factors IFN-β, IFN-γ, ISG15, Mx1, and OAS1 were significantly upregulated in the intestine of mice in the vaccination group compared with the vehicle group, and the antiviral factors were significantly increased in the vaccination and vehicle groups compared with the mock group (Figure 4G).

## Discussion

The PRV variant strains exhibited stronger neurotropism and more serious neuropathological lesions than the PRV classical strains (Luo et al., 2014; Wang et al., 2019b). Many human encephalitis cases were reported (Wang et al., 2019a; Li et al., 2020). The patients started with a high fever, headache, and rapid progression to signs of a central nervous system infection, including altered mental status, seizures, and coma (Yang et al., 2019). Vaccination is still the most efficient way to prevent and control PRV, especially gene-deleted vaccines. The gE target infects second- and third-order neurons of the olfactory and trigeminal routes, and gI affects the anterograde-directed transport of virus in neurons and the spread in non-neuronal cells (Kratchmarov et al., 2013). TK is involved in the viral replication and neuroinvasiveness of PRV in the central nervous system (Kit et al., 1985). TK deficiency significantly reduced the ability of replication and transmission in nerve cells (Lv et al., 2021). As the classical model animals for PRV research, mice were used in our research. In this study, we constructed a gE/gI/TK-deleted strain and explored the vaccine protection of the central nervous system in mice. The data suggested that PRV XJ delgE/gI/TK was an effective tool strain for investigating neurological damages and a promising vaccine candidate for PRV prevention and control.

It has been reported that inflammation of the central nervous system exacerbates the blood–brain barrier permeability and reduces tight junctional protein expression (McColl et al., 2008). In many nervous system diseases, secondary pathophysiological changes occur in the central nervous system after blood–brain barrier injuries, such as brain edema, elevated intracranial pressure, and internal environment disorder, further aggravating the neurological damage (Laval et al., 2018; Sun et al., 2018; Jiang et al., 2021; Jiao-Yan et al., 2021). Our data showed no blood–brain barrier leakage after the PRV challenge. The blood–brain barrier was not destroyed after the PRV challenge because PRV cannot induce viremia. In addition, the viral loads and inflammation were significantly decreased, and no neurological damages were observed in the vaccination group compared to the vehicle group. Yeh proved that PRV infection increases TNF-α expression, and TNF-α is a key mediator in PRV-induced apoptosis *in vitro* (Yeh et al., 2008), so we speculated that PRV XJ delgE/gI/TK protected the central nervous system from neurological damage by inhibiting inflammation.

Because the gE, gI, and TK deletion PRV strain cannot transport to the central nervous system, the vaccine cannot activate the immunity of the central nervous system by a direct effect with the central nervous system immune cells, including astrocyte, microglial cells, and oligodendrocyte (Pomeranz et al., 2005; Klein et al., 2017). Sun verified that the antiviral factors IFN-β and IFN-γ were upregulated at the early phases of PRV infection and decreased at later periods (Sun et al., 2021). In our research, the reason why ISG15, Mx1, OAS1, IFN-γ, and IFN-β in the brain showed no difference between the vaccination group and the vehicle group might be that the mice were in a later period of infection. In our studies, ISG15, Mx1, OAS1, IFN-γ, and IFN-β in the intestine of the vaccination group were increased compared with the vehicle group. Maybe it is because PRV infection would induce a lot of antiviral factors in the intestine where a variety of immune cells are located, and gE, gI, and TK deletion PRV could induce a higher IFN-β than wild PRV (Lv et al., 2021). Furthermore, the pro-inflammatory factors TNF-α and IL-6 were downregulated in the vaccination group, in the brain and intestine, compared with the vehicle group.

In summary, inflammation was inhibited and intestinal immunity was strongly activated in the vaccination group. Recent studies have demonstrated that gut immune-stimulatory products can influence microglia function to prevent central nervous system damage following a viral infection (Brown et al., 2019). In our study, we found that the PRV XJ has no effect on the intestine, except the intestinal villi height and growth, of the vaccination group compared with the vehicle group (Supplementary Figure 1). Therefore, PRV XJ del gE/gI/TK protected the central nervous system from PRV-induced neurological damages by inducing intestinal immunity. We speculate that PRV XJ del gE/gI/TK induced central nervous system immunity through the brain–gut axis, and our laboratory has developed a related research.

## Data Availability Statement

The original contributions presented in the study are included in the article/Supplementary Material, further inquiries can be directed to the corresponding author.

## Ethics Statement

The animal study was reviewed and approved by the Sichuan Agricultural University Ethics Committee.

## Author Contributions

LX, J-FW, and LZ contributed to conceptualization. LX and J-FX contributed to methodology. LX and JZ contributed to software and writing—original draft preparation. Z-WX contributed to validation. LX, F-QL, and S-YX contributed to investigation. Y-CZ and Z-WX contributed to resources. LX contributed to data curation and writing—original draft preparation. M-CN contributed to supervision. LZ contributed to project administration and funding acquisition. All authors have read and agreed to the published version of the manuscript.

## Conflict of Interest

The authors declare that the research was conducted in the absence of any commercial or financial relationships that could be construed as a potential conflict of interest.

## Publisher’s Note

All claims expressed in this article are solely those of the authors and do not necessarily represent those of their affiliated organizations, or those of the publisher, the editors and the reviewers. Any product that may be evaluated in this article, or claim that may be made by its manufacturer, is not guaranteed or endorsed by the publisher.

## Footnotes

1
https://zlab.bio/guide-design-resources

## Supplementary Material

Supplementary Figure 1 Histopathological observations of mice intestine injected with the indicated viral strain or Dulbecco’s modified Eagle’s medium (hematoxylin and eosin staining, ×200 magnification).
