# Supplementary material for: The Immunity Protection of Central Nervous System Induced by Pseudorabies Virus DelgI/gE/TK in Mice
Source: Front Microbiol. 2022 Mar 25;13:862907. doi: 10.3389/fmicb.2022.862907 (PMC8990752; doi:10.3389/fmicb.2022.862907)
Supplement: Supplementary Figure 1 — Histopathological observations of mice intestine injected with the indicated viral strain or Dulbecco’s modified Eagle’s medium (hematoxylin and eosin staining, ×200 magnification). [file Image_1.pdf]

Supplementary material:

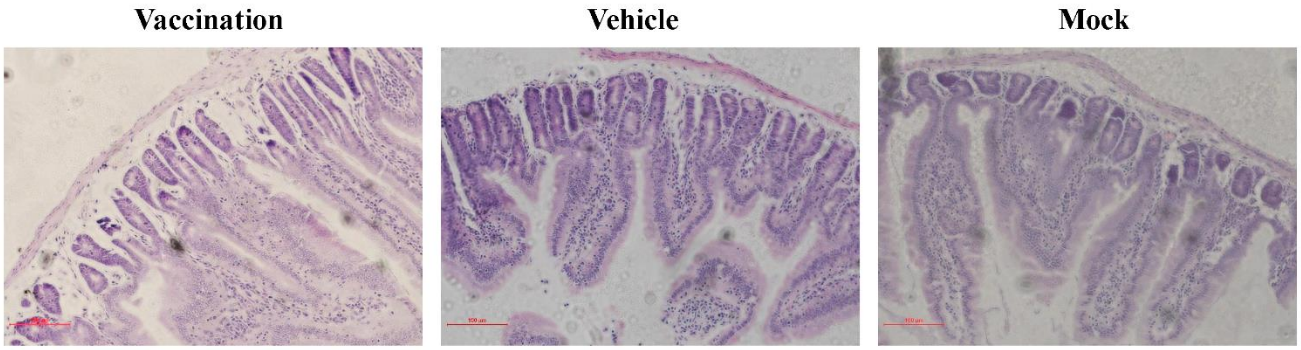

Fig.S1. Histopathological observations of mice intestine injected with the indicated viral strain or DMEM. (hematoxylin and eosin staining, 200×magnification)
